# Computational Simulation Study-Based Formulation Development and Characterization of MethylprednisoloneLoaded Nanoparticles Containing Chitosan and Pectin to Treat Nocturnal Asthma

**DOI:** 10.3390/polym17010024

**Published:** 2024-12-26

**Authors:** Vijaya Kumar Voleti, Ismail Yusuff, Mohamed Jalaludeen Abdulkadhar, Mohammad Khalid Al-Sadoon

**Affiliations:** 1 Crescent School of Pharmacy, B. S. Abdur Rahman Crescent Institute of Science and Technology, GST Road, Vandalur, Chennai 600048, Tamil Nadu, India; vijay66vvk@gmail.com; 2 Crescent Global Outreach Mission Research and Development, B. S. Abdur Rahman Crescent Institute of Science and Technology, GST Road, Vandalur, Chennai 600048, Tamil Nadu, India; mohamedjalaludeen@crescent.education; 3 Department of Zoology, College of Science, King Saud University, P.O. Box 2455, Riyadh 11451, Saudi Arabia; msadoon@ksu.edu.sa

**Keywords:** nocturnal asthma, computational study, methylprednisolone, chitosan, chitosan succinate, chronomodulated delivery system, histopathological study

## Abstract

Nocturnal asthma (NA) is a high-prevalence disease that causes severe respiratory issues, leading to death from early midnight to early morning. In this research, nanoparticulate drug delivery system of methylprednisolone (MP) was developed using chitosan (CH) and pectin (PEC). MP is a synthetic corticosteroid medication widely used for its potent anti-inflammatory activity. Computational simulation study (AI-based blend analysis algorithm) was used to identify a better-mixing polymer with MP. MP nanoparticles were formulated by the ionic gelation method with the combination of CH and PEC. To modify the drug release properties, the formed beads were coated with chitosan succinate (CSSC). The morphological characteristics of the beads were determined by SEM analysis. The X-ray radiographic imaging study was used to observe the intactness of MP beads. Histopathological studies were also carried out to find out the toxicity of the beads in the organs of rats. Pectin and chitosan polymers were selected based on the computational simulation study. SEM analysis revealed that the beads had a spherical shape with a rough outer surface. CSSC-coated beads achieved sustained drug release for up to 24 h. X-ray imaging demonstrated the stability of the beads in acidic pH conditions. In vivo pharmacokinetic studies showed that CSSC-coated beads were more stable in the gastrointestinal tract (GIT) than PEC-CH beads and the pure drug. Histological evaluation confirmed that the beads are nontoxic and safe for use in rats. Based on the findings, it was concluded that CSSC-coated beads of MP exhibited superior release properties, making them suitable for a chronomodulated drug delivery system.

## 1. Introduction

Asthma is a chronic respiratory condition that affects people globally, with significant variability in prevalence, morbidity, and mortality rates across regions. The following are some key global statistics on asthma. Approximately 339 million people worldwide suffer from asthma according to the Global Asthma Report by the Global Initiative for Asthma (GINA) and the World Health Organization (WHO). Asthma accounts for around 455,000 deaths annually, as reported by WHO [1,2].

NA is characterized by a distinctive worsening of symptoms during the night, particularly between 3:00 a.m. and 7:00 a.m. This aggravation is linked to the body’s circadian rhythms, which affect airway inflammation and lung function. Consequently, these rhythms lead to heightened airway resistance and reduced pulmonary function during these early morning hours [3]. Patients with NA often experience a sharp decline in peak expiratory flow (PEF) during this period, which leads to wheezing and sleep disturbances. Conventional asthma treatments, which rely on sustained drug release, often fail to provide adequate control over these nocturnal exacerbations [4,5]. Therefore, targeted drug delivery systems that align drug release with the circadian rhythms of NA are crucial for improving therapeutic outcomes and the patient’s quality of life.

Polymers are crucial in various drug delivery methods and formulations, including oral, transdermal, and implantable systems. Their adaptability allows for customized release kinetics, from immediate to sustained or pulsatile release, enhancing therapeutic efficacy and patient compliance. Recent studies demonstrate that polymers can be precisely tailored for specific delivery patterns, as shown in research on innovative drug delivery approaches [6]. This broad range of applications underscores the potential for polymer-based systems to explore new therapeutic areas and administration routes, meeting diverse clinical needs.

MP, a potent anti-inflammatory corticosteroid, is frequently used to manage chronic inflammatory conditions like asthma. According to the Biopharmaceutics Classification System (BCS), MP is categorized as class II drug, having hydrophobic (log *p*-2.58) properties with limited water solubility [7]. A novel approach to mitigate these issues involves encapsulating MP within a biodegradable nanocarrier system capable of delivering the drug in a controlled, chronomodulated fashion. Pelletized multiparticulate dosage forms (such as pellets), considered a better choice, proved themselves as a promising dissolution enhancer for BCS class II drugs [8]. This gives a basic idea for developing chitosan succinate-functionalized pectin–chitosan nanoparticle beads (MP-CSSC-PEC-CH Beads) for chronomodulated delivery of MP for the management of NA.

By timing the release of MP to coincide with the early morning hours when NA symptoms are most severe, the therapeutic efficacy can be enhanced while minimizing adverse effects. Computational studies were employed to offer a conceptual framework for choosing the optimal polymer for formulation. In recent years, chitosan-based nanoparticles have attained considerable attention as carriers for drug delivery systems due to their biocompatibility, biodegradability, and ability to protect drugs from premature degradation in the gastrointestinal tract. Chitosan is a natural polysaccharide that provides a stable matrix for encapsulating hydrophilic and hydrophobic drugs alike [9,10]. Moreover, the mucoadhesive properties of chitosan allow for prolonged drug retention at the absorption site, increasing bioavailability and ensuring a sustained therapeutic effect. The development of chitosan-based nanoparticles for MP offers the potential to create a stable and targeted delivery system tailored for the treatment of nocturnal asthma [11].

The objective of this study is to synthesize and characterize a stable chitosan-based nanoparticulate delivery system for MP, designed specifically for the chronomodulated treatment of NA. The nanoparticles are formulated to release MP in a pulsatile manner, ensuring that the drug is available during the critical hours of early morning when asthma exacerbations are most likely to occur. In vitro characterization, including particle size, drug encapsulation efficiency, and release kinetics, was conducted to evaluate the stability and efficacy of the synthesized nanoparticles. In order to achieve the goals, we created a potent formulation and tested it using analytical techniques such as Fourier transform infrared spectroscopy (FTIR), scanning electron microscopy (SEM), and X-ray analysis to assess the movement of the beads through GIT. We then optimized the formulation’s in vitro swelling index at both basic and acidic pH levels, as well as its in vitro drug release of MP. Rat models were used to study the effects of toxicity tests on different organs, including the kidney, heart, and lung, as well as in vivo pharmacokinetics characteristics.

## 2. Materials and Methods

### 2.1. Materials

MP was received as a gift sample from Madras Pharmaceuticals, Chennai. Chitosan ((>75%), deacetylation and viscosity of 20–300 cps (1% in 1% acetic acid)) was procured from Sigma Aldrich, Saint Louis, MO, USA. Pectin (DE  >  50%) was procured from SD Fine Chemicals, MH, India. We bought calcium chloride and succinic anhydride from Merck, KA, India. Unless otherwise noted, all other reagents utilized in the investigations were of analytical quality. Throughout the investigation, double-distilled water from the in-house facility was used.

### 2.2. Selection of Polymers by AI-Based Blend Analysis Algorithm

This study sought to predict the mixing energy and atomic-level interactions between MP and a range of polymers, such as starch, sodium alginate, pectin, lactose, hydroxypropyl methylcellulose, ethylcellulose, chitosan, and carboxymethyl cellulose. Computational simulations were employed to offer a conceptual framework for choosing the optimal polymer for formulation [12].

#### 2.2.1. Theoretical Simulation Software

The physicochemical profiles of the drug and polymer components were assessed using two simulation platforms: “BIOVIA” Material Studio (V-23.1.0.3829) and Discovery Studio 2021 (Version 21.1.0.1643). These tools were utilized to visualize the interactions between the polymers and bioactive molecules

#### 2.2.2. Ground State Energy Level of the Components

The molecular mechanics (MM) tool, Forcite, was used to optimize the energy of each formulation component. For energy level analysis, 2D structures in the structure data file (SDF) format of the drug (MP) and polymers such as starch, sodium alginate, pectin, lactose, hydroxypropyl methylcellulose, ethylcellulose, chitosan, and carboxymethyl cellulose were input into the Forcite protocol. The Condensed-phase Optimized Molecular Potentials for Atomistic Simulation Studies (COMPASS) algorithm was employed to calculate the potential energy of the formulation components by summing the Van der Waals and electrostatic properties of each atom, with other parameters set to default values.

#### 2.2.3. Blend Mixing and Adsorption Location Between the Drug and the Polymer

The blend algorithm was used to calculate the mixing energies and average mixing energies of MP with each polymer in the formulation. It assessed pairwise energies using different trajectories and an energy bin width of 0.020 kcal/mol for atom-based nonbonded interactions. The final **E**_mix_ energy was determined by summing these interactions [13]. E-mix energy was calculated using the following equation (Equation (1)).

(1) $$E_{\mathrm{mix}\;}=\frac{1}{2}\left(Z_{bs}{\left[E_{bs}\right]}_{T}+Z_{sb}{\left[E_{sb}\right]}_{T}-Z_{bb}{\left[E_{bb}\right]}_{T}-Z_{ss}{\left[E_{ss}\right]}_{T}\right)$$

Following the blend mix energy calculation, the minimized components were input into the adsorption location identification module. The atomic-level interactions (bonded, nonbonded, and non-favorable atom-based) were then visualized using the Biovia Material Studio 2023 (V-23.1.0.3829) and Discovery Studio 2021 (Version 21.1.0.1643).

### 2.3. Preparation of MP-Loaded Chitosan Nanoparticles

The required quantity of chitosan (100 mg) was mixed with the 1% aqueous acetic acid solution (50 mL in quantity). MP was dissolved in ethanol. The prepared ethanolic MP solution was slowly poured into previously prepared chitosan solution with continuous stirring for about 30 min. The resultant solution was subjected to warming at a temperature of 60 °C for about 10 min. TPP was dissolved in water and 33 mg of TPP was used for 100 mg of chitosan. The ratio of chitosan to TPP was (3:1). To this warmed mixture of MP–chitosan solution, 1 mg/mL sodium tripolyphosphate (TPP) was added dropwise and the solution was stirred (700 rpm). After the complete addition of TPP, the solution was stirred continuously for another hour to aid the polyionic complexation at room temperature. Then, to separate the nanoparticle thus formed, centrifugation at 7500 rpm for 30 min was used (Remi Model Number: R-8C, Mumbai, India, followed by washing with deionized water and then drying [14,15].

### 2.4. Preparation of Chitosan Succinate Polymer

The preparation of the chitosan succinate polymer was carried out using the method previously reported [16,17,18]. The synthesis of chitosan succinate was carried out using the method of replacing primary amino groups with other substituents. Briefly, chitosan (1.00 g, corresponding to approximately 6.20 mmols glucosamine) was dissolved in an aqueous solution of hydrochloric acid (HCl) (0.37%, 50 mL) at ambient temperature, and a solution of the anhydride (6.25 mmol; succinate 0.63 g) in pyridine (5 mL) was added dropwise with vigorous stirring. The reaction pH was maintained at 7.0 by the dropwise addition of 1M sodium hydroxide (NaOH) solution. After 45 min the reaction was terminated by the addition of sodium chloride (NaCl) solution (20%, 200 mL). The resulting precipitate was filtered and washed with acetone and diethyl ether. The polymer was stored in a desiccator and used for further studies.

### 2.5. Preparation of CSSC-Coated MP-Loaded Chitosan Beads

CSSC was prepared and the yield was found to be 90%. The average degree of chitosan substitution by succinate moieties was found to be 12.3%. CS is soluble in aqueous medium. To sum up, 20 mL of MP-loaded CH nanoparticles was dissolved in a PEC solution, and the resulting mixture was introduced into a 10% calcium chloride solution using a syringe and a needle (ID = 2 mm) while stirring. PEC immediately gelled upon coming into contact with Ca^2+^, producing beads. The beads were agitated for 30 min before being filtered and freeze-dried. A previous study by our group was followed in the production of CSSC polymer. Specifically, 2.5% *w*/*v* CSSC solutions in distilled water were gradually added to MP-loaded PEC-CH beads while being stirred magnetically at room temperature (100 rpm) for approximately two hours [16,17]. The pH was adjusted to pH 5 before being stored overnight at 4 °C in the refrigerator [18]. The resulting product was MP-loaded CSSC-PEC-CH beads (Table 1).

### 2.6. Determination of Drug Entrapment Efficiency

The formulated CSSC-PEC-CH beads of about 100 mg were powdered well and immersed in 10 mL of ethanol overnight, then sonicated for 15 min. The resulting solution was filtered using No. 40 Whatman^®^ filter aid to eliminate undissolved polymer debris [19]. The filtrate was appropriately diluted with phosphate buffer (pH 7.4) and the drug content was measured at 247 nm by using (a Shimadzu UV-VIS spectrophotometer, Model Number: UV-1800, Tokyo, Japan). The drug entrapped into the beads was derived with equation (Equation (2)):

(2) $$E\%\;=\;\frac{Actualdrugcontentinbeads}{\mathrm{Theoreticaldrug}\;\mathrm{content}\;\mathrm{in}\;\mathrm{beads}}\;\times 100\;$$

### 2.7. Determination of the Surface Morphology and Size of the Formulated Beads

The size and form of the prepared beads were assessed using a Nikon DS-Fi 20 digital microscope. The size of the beads was measured using a digital vernier caliper. Similarly, the surface morphology of the beads was examined using SEM (JEOL, model number: JSM 6390LV (Tokyo, Japan). To improve the particle’s conductivity, gold sputter coating was applied using a vacuum ion sputter for 75 s after the beads were mounted on an SEM stub with the help of double-sided adhesive tape [20].

### 2.8. FTIR Analysis for MP and Other Excipients

The beads and particles were subjected to FTIR (Perkin Elmer, Model Number: L160000A, Waltham, MA, USA) analysis using KBr-pressed pellets. Every finely ground sample was combined with KBr (1:9) and formed into a pellet using a 100 kg hydraulic press. A spectral scanning from 4000 and 400 cm^−1^ wavenumber was then performed.

### 2.9. Evaluation of the Swelling Index

Swelling studies of CSSC-PC-CH nanoparticle beads were carried out at (37 ± 0.5) °C at pH 1.2 (acidic) and pH 7.4 (phosphate buffer), respectively. Periodically, the soaked beads were removed, the excess media was blotted off using blotting paper, and the beads were weighed [21]. The following formula (Equation (3)) was then used to determine the swelling index (%).

(3) $$Swelling\;Index\;=\;\frac{Wt-Wo}{\mathrm{Wo}}\;\times 100$$

where *W*_t_ is refers to the weight of beads at time *t*, and *W*_0_ refers to the weight of the beads before soaking them into specific media.

### 2.10. In Vitro Analysis of Drug Release Studies from MP-CSSC-PC-CH Nanoparticle Beads

In vitro release performance of CSSC-PEC-CH nanoparticle beads was performed using USP dissolution-I basket (Electrolab, model number: PSBSK040-EL, Mumbai, India) at 37 ± 1 °C and puddling speed of 100 rpm. An accurate quantity of MP-loaded CSSC-PEC-CH equivalent to 50 mg MP was added to 900 mL of dissolution medium (0.1 N HCl) for about 2 h and then dissolved in phosphate buffer (pH 7.4) for further treatment [22]. Approximately 5 mL aliquots of the solution were collected at specified time intervals, and after each collection, an equal amount of fresh specific pH buffer was added to the dissolution vessel to maintain the same settling conditions. The collected samples were filtered and appropriately diluted to measure the amount of drug at 247 nm [23]. Three measurements were considered for each sample by repeating the experiment.

### 2.11. X-Ray Radiographic Imaging Analysis of MP-CSSC-PC-CH Nanoparticle Beads

X-ray imaging technique was used to monitor the presence of the nanoparticles throughout the GI system. This can be achieved by the incorporation of the radio-opaque material (Barium Sulphate) in place of the drug into the formulated nanoparticles. Then, the nanoparticles were administered through an oral route to the overnight-fasting albino rats using plastic tubing and then flushing with 10–15 mL of water. An abdominal X-ray imaging at 20 mA, 55 kV, and 8 mAs (Genius-60 Mobile portable unit, Wipro GE) in a sleeping position was carried out at different time intervals after anesthetizing rats using ketamine hydrochloride (20 mg/kg dose). By this method, we could observe the intactness of chronomodulated products before elimination. The institutional ethical committee approved the protocol of the X-ray imaging study [24,25].

### 2.12. In Vivo Pharmacokinetic Studies

Male Wistar rats weighing 220–250 g were acquired from Santhiram College of Pharmacy Animal House in Andhra Pradesh, India. The purchased rats were housed in a typical laboratory setting with 25 ± 1 °C and 55 ± 5% relative humidity. The rats were kept in polypropylene cages and fed a standard laboratory diet (Lipton feed, Mumbai, India) with unlimited access to water. The CPCSEA protocol (approval number 1519/PO/Re/S/11/CPCSEA/2023/002) requirements had been followed throughout the investigation [26].

Six-rat groups were given oral doses of MP suspension: single dosage (20 mg/kg), PEC-CHNP beads, and CSSC-PEC-CHNP beads. Blood samples from rats were collected into tubes containing EDTA at 0, 1, 2, 4, 6, 8, 10, 12, 16, 20, and 24 h; the plasma was separated immediately by centrifugation at 3000 rpm for 15 min, and it was then kept at −20 °C until analysis. With just minor adjustments, the previously published HPLC technique was used to estimate the amount of MP in the samples [27]. The MP plasma concentration (Vs) time plot was obtained using the non-compartmental analysis model. The experimental parameters, such as T_max_ and peak plasma concentration (C_max_), were immediately determined. Additionally, a log-linear regression analysis of the plasma concentration (Vs) time plot was used to determine the elimination rate constant (k_el_). Using the k_el_, the elimination half-life (t_1/2_) was also determined. Using the same concentration (Vs) time plot from 0 h to time-t, the last measurable concentration observed, the area under the curve (AUC_0–t_) was computed using the linear trapezoidal method [28]. By dividing the final plasma concentration that could be measured by the elimination constant (K_e_), the AUC_t–∞_, or concentration at infinite time, was computed. Additionally, the relative bioavailability and the mean residence duration were assessed (MRT) [29,30].

### 2.13. Stability Assessment of MP CSSC-PC-CH Nanoparticles

The optimized batch’s beads were put in HDPE bottles, sealed, and kept in a stability chamber (Chennai, India) for six months at 25 °C/60% RH and 40 °C/75% relative humidity in compliance with ICH regulations. Samples were taken out after six months, and their physical attributes, drug content (assay), bead size (mm), and test results for the lag-timed release of the medication (T lag) were analyzed.

## 3. Results and Discussions

### 3.1. Forcite Minimization Energy Profile of Mixing Components

**Figure 1:**
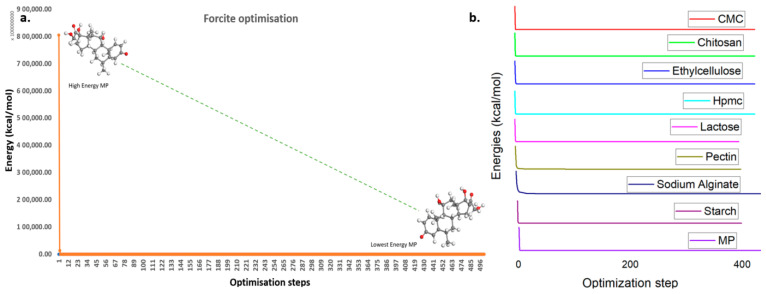
Geometric optimization: (**a**) geometric optimization of methylprednisolone; (**b**) geometric optimization of the polymer.

The Forcite module algorithm results included four parameters: total energy (initial and final conformational structures) and RMS force (initial and final conformational structures) (Table 2). This table also shows the transition of the molecule’s energy from an excited state to a lower energy (ground state), leading the components to achieve a stable configuration (Figure 1). From the geometric optimization energy profile of the components (Figure 1), it was observed that the energy of MP initially started at 80,572,880.0 kcal/mol and decreased to 85.9 kcal/mol. The molecule achieved a stable configuration through torsion angle modification and side fragment angle modification. Similarly, the energies of all polymer components were minimized to local energy minima (Table 1). The geometric optimization energy graph indicated that energy reduction began at the 4th interaction and then stabilized at the local minimum energy level (Figure 1). Sodium alginate and carboxymethyl cellulose energies fluctuated during the 10–25 optimization steps and formed stable conformation at the 25th optimization step. The energies of all other polymers were also minimized and subjected to interaction analysis.

### 3.2. Blend Mixing of Various Polymers and the Drug

The binary mixture compatibility was assessed using the Flory–Huggins model and molecular simulation techniques. The blend protocol provided χ values for mixtures of MP with various polymers, including starch, sodium alginate, pectin, lactose, HPMC, ethyl cellulose, chitosan, and carboxymethyl cellulose. The results indicated that the MP–pectin mixture had the most favorable interaction, with mix energies of −17.81168398 kcal/mol and −9.44170033 kcal/mol. Generally, a negative or small energy value suggests favorable interactions at this specific temperature, indicating that the two molecules will form a single-phase mixture.

**Figure 2:**
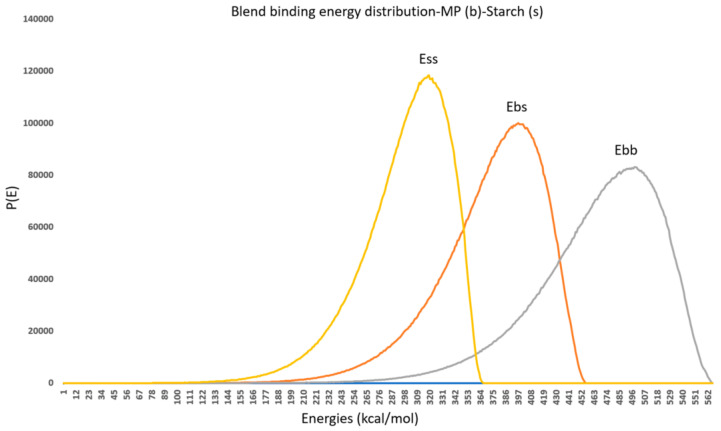
The Base (MP)–Base (MP) (grey), Base (MP)–Screen (polymer) (orange), and Screen (polymer)–Screen (polymer) (yellow).

**Figure 3:**
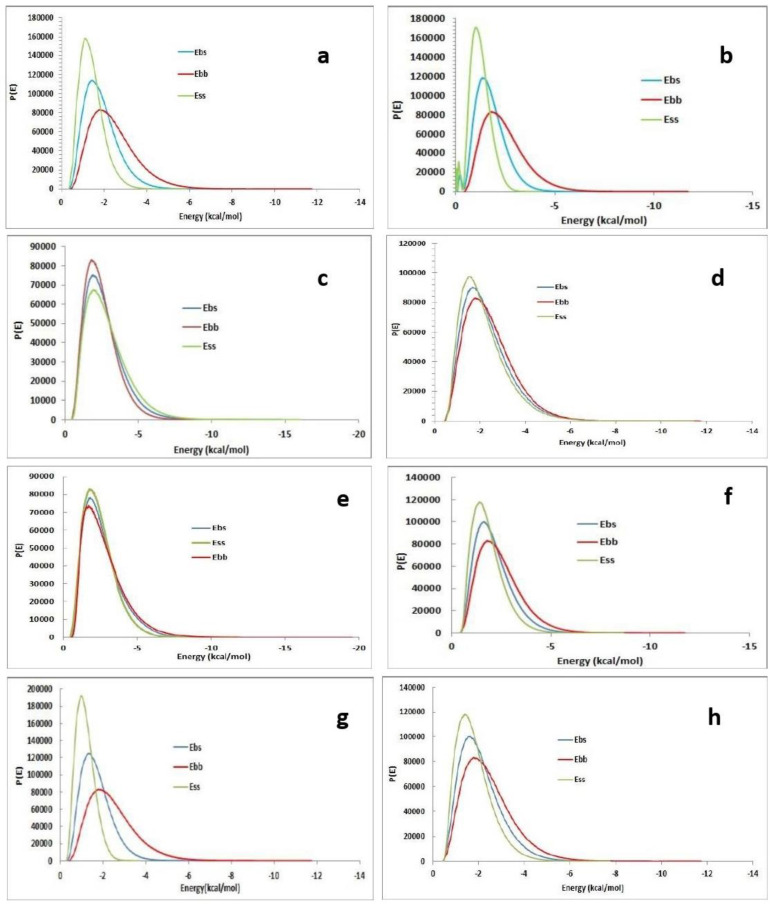
Blend binding energy distribution of (**a**)MP–starch; (**b**) MP–sodium alginate; (**c**) MP–HPMC; (**d**) MP–lactose; (**e**) MP–pectin; (**f**) MP–ethyl cellulose; (**g**) MP–chitosan; (**h**) MP–carboxymethyl cellulose.

The possible combinations of the drug and polymers were illustrated as Base–Base (grey), Base–Screen (orange), and Screen–Screen (yellow) combinations graphically (Figure 2 and Figure 3). From the blend mixing module result, it was observed that the MP and HPMC combination was mixed well, with a stable negative energy of −9.441 kcal/mol (Figure 3e). Other combinations of energies show higher differences, indicating the immiscibility of the drug. This indicates the MP–HPMC formulation acted better as a drug-delivery formulation.

#### 3.2.1. Interaction Pattern and Interpretation

**Figure 4:**
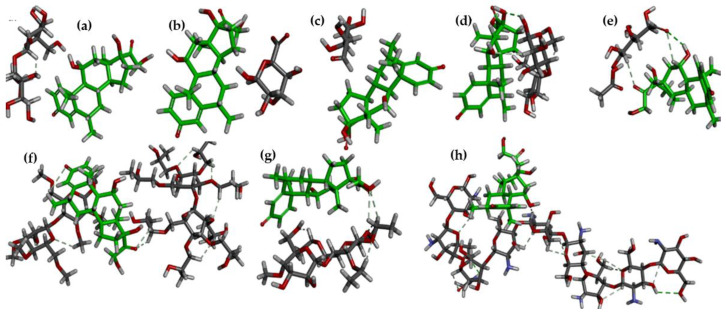
Adsorption location analysis of the drug and polymer. (**a**) MP–starch; (**b**) MP–sodium alginate; (**c**) MP–HPMC; (**d**) MP–lactose; (**e**) MP–pectin; (**f**) MP–ethyl cellulose; (**g**) MP–chitosan; (**h**) MP–carboxymethyl cellulose (green-MP).

**Figure 5:**
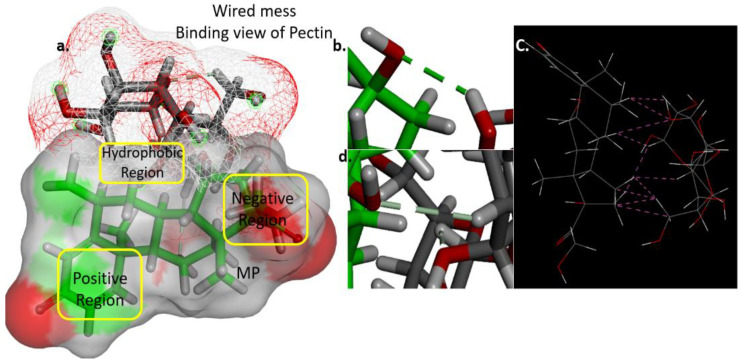
Pectin binding interaction with MP drug molecule. (**a**) Pectin (wired surface) binding on MP (Solid surface). (**b**) Hydrogen bonding of pectin and MP. (**c**) Close contacts of pectin and MP. (**d**) Conventional hydrogen bond pectin and MP.

MP and all the polymer interaction patterns were illustrated in Figure 4 and Figure 5, which provide the adsorption atom locations between these polymer and drug molecules. Among the eight polymers, four generate interactions with MP.

Figure 5 visualization is likely used to understand how different molecular regions interact, which is essential in fields like drug design, and illustrates the binding atoms of pectin and MP, specifically two conventional hydrogen bonds generated. The hydroxy (-OH) group in the ring and the aliphatic chain of MP networked with the hydrogen atoms of the pectin polymer (Figure 5b,d). The surface binding of MP and pectin shows the hydrophobic attraction and electronegative glaze (Figure 5a). MP binds well in the polymer cavity and traps the drug molecule with a good affinity [31]. Close contact calculation showed that 10 interactions strengthen the molecule and make the complex a sustained release formulation. As another polymer, chitosan forms a good mixing energy of −11.48541631 kcal/mol and an adsorption energy of −55.169463 kcal mol with MP.

#### 3.2.2. Entrapment Efficiency

When PEC was added to a calcium chloride solution, a gel-like structure initially developed. This structure changed into a robust bead when the string was continuous. Higher degrees of cross-linking during the last phases of bead production are the cause of this. This very cross-linked structure is likely to increase drug entrapment efficiency by lowering drug loss to the CaCl_2_ medium. This was comparable to other research on the interpenetrating polymeric network between chitosan and PEC.

### 3.3. Determination of Surface Morphology and Size of the Formulated Beads

**Figure 6:**
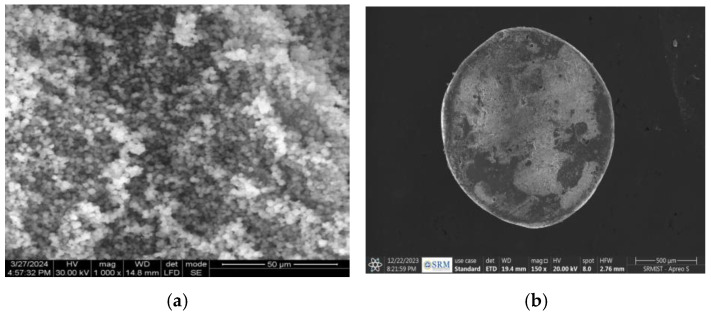
SEM images of the (**a**) MP-loaded chitosan nanoparticles and (**b**) CH-PEC beads formulation.

The MP-loaded chitosan nanoparticles were prepared using the ionic gelation mechanism. The SEM image of MP-loaded nanoparticles is presented in Figure 6a. The nanoparticles form due to ionic interactions between chitosan and TPP, which cause the chitosan molecules to cross-link. The gradual addition of TPP ensures controlled cross-linking, preventing aggregation and yielding stable nanoparticles. The size and shape of the beads vary depending on the PEC concentration since those factors only affect the number of sites that are available for cross-linking with Ca^2+^ ions. The shrinkage of polymeric gel beads at greater cross-linking concentrations provides compelling evidence for this. SEM microscopical analysis was used to study the morphology of MP-entrapped optimized CSSC-PEC-CH nanoparticle beads (Figure 6b). The size of the beads was found to be 1022 ± 29 μm. According to the data, the CSSC-PEC-CHNP beads revealed a spherical form with a rough surface at the outer phase and tiny fissures and cracks that resembled networks. These features may have originated from water molecules escaping, which created a porous outer network and caused the bead structure to shrink [32]. The lack of drug crystals on the bead surface indicated a fine and molecular dispersion of the drug throughout the PEC-CH matrix.

### 3.4. FTIR Analysis for MP and Other Excipients

**Figure 7:**
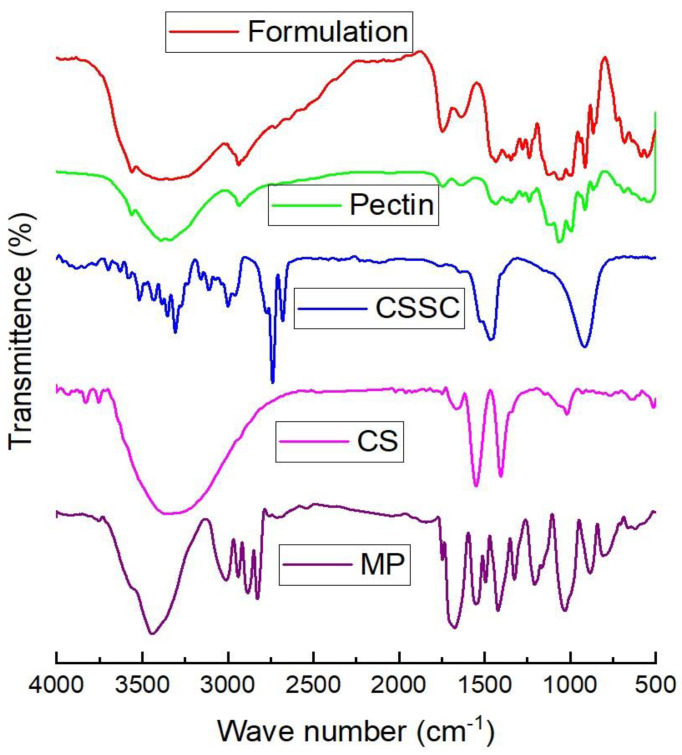
FTIR spectra of MP, PEC, CH, CSSC, and MP-loaded bead formulation.

Figure 7 portrays spectra of MP, PEC, CH, CSSC, and MP-loaded bead formulation. In the FTIR spectra of MP, there were three distinct peaks that indicated C=O (ketone) at 1715.7 cm ^−1^, C=C (alkenes) at 1650–1550 cm^−1^, and O–H (alcohol) at 3400–3100 cm^−1^. The FTIR spectra of CSSC revealed that −NH-C=O stretching band arises in the range of 1637–1659 cm^−1^ and carboxylic C=O stretching was observed in 1703–1734 cm^−1^. The new vibrational band formed at 1722 cm^−1^ corresponds to the carbonyl group of an ester (C=OR), while the band for the acyl amino -CO group was observed at 1647 cm^−1^. This result evidences the effective insertion of the succinic group into the amino and hydroxyl groups of the chitosan backbone. Pectin contains multiple galacturonic acid units linked by alfa-(1–4) linkage, confirmed by the FT-IR absorption spectrum peaks. Additionally, the ketonic groups, OH, C-O-C, and alkyl groups peaks were found at 1700 cm^−1^, 3400 cm^−1^, 1100 cm^−1^, and 2800 cm^−1^, respectively. C-O stretch: around 1000–1300 cm^−1^, indicating C-O single bonds in the sugar units. The best-combined formulation loaded with MP showed broadened peaks. O-H stretch: around 3200–3600 cm^−1^, indicating the presence of hydroxyl groups. C-H stretch: around 2800–3000 cm^−1^, corresponding to aliphatic C-H stretching. C=O stretch: around 1700 cm^−1^, representing carbonyl groups (ester linkages). C-O stretch: around 1000–1300 cm^−1^, indicating C-O single bonds. The FTIR spectra of the MP-loaded CSSC-PC-CH bead formulation showed all the characteristic peaks of MP, indicating that there is no interaction between the polymer and the drug.

### 3.5. Determination of the Swelling Index

It was observed that coating the PEC-CH beads with CSSC imbibes the swelling index in acidic pH, which hinders the gel porosity. This supports the protection of the drug from the harsh acidic environment of the stomach. The reason behind this is the cross-linking of the Ca^2+^ ions by the Cl^−^ ions of the gastric fluid, which remained unionized, and the poor charge repulsions. This effect was opposite of the case of pH 7.4, in which the swelling index of the beads significantly enhanced along with PEC concentration due to the basic condition that induces more -COOH to get ionized and exhibit more hydrophilic properties at this pH.

### 3.6. In Vitro Drug Release Studies of MP-CSSC-PC-CH Nanoparticle Beads

**Figure 8:**
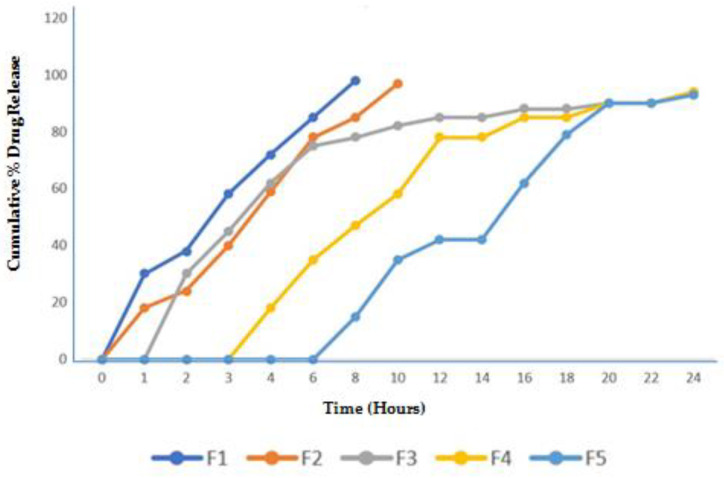
The effect of CSSC coating on the in vitro dissolving profile of MP-loaded PEC-CH beads at different pH levels (1.2 and 7.4).

From the in vitro release studies (Figure 8), it was observed that in the acidic medium, a notable and sustained drug release was observed up to 12 h, with around 20% of the drug released after 2 h. In the alkaline medium, PEC-CH beads showed a faster release of MP. The findings showed that pectin and chitosan may potentially interact through hydrogen bonding at low pH levels (pH < 2), increasing the hydrophilic qualities of the beads. Because of the robust matrix structure that developed between the positive charge of chitosan and the negative charge of PEC, MP release was delayed in PEC-CH beads. However, pulsatile pattern drug release was detected from the beads coated with the CSSC polymer. The outcome can be explained by the fact that the CSSC polymer backbone’s COOH group is predominantly in a unionized state in acidic environments (pH 1.2), making them weakly hydrophilic, hence the poor release at low pH; whereas, in the instance at pH 7.4, ionization was maximum, with a high degree of swelling, and therefore, the diffusional path length of the drug was greatly increased [30].

### 3.7. X-Ray Radiographic Imaging Studies of MP-CSSC-PC-CH Nanoparticle Beads

**Figure 9:**
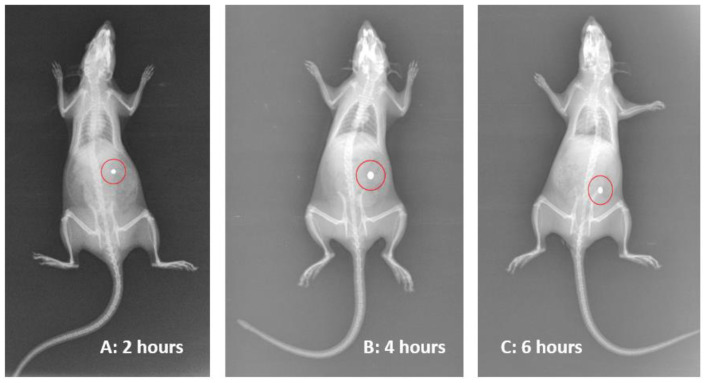
X-ray images of chronomodulated CSSC-PEC-CH beads of MP in rats, (**A**) after 2 h, (**B**) after 4 h, and (**C**) after 6 h.

X-ray scintallography studies (Figure 9) were performed to elucidate the in vivo mobility nature of optimized chronomodulated CSSC-coated PEC-CH beads in albino rats. From the images, it was found that, after the administration of the beads, the beads slowly reached the intestinal region after 4 h of the study. This revealed the intactness of the chronomodulated beads in the stomach and small intestine. At the end of the 6th hour, barium sulfate was released from the beads into the small intestine by the swelling and erosion of the CSSC. Due to the mucoadhesive nature of the PEC-CH polymer, some remaining debris of the beads adhered to the small intestinal region. The results of the in vivo X-ray study were supported by the in vitro dissolution studies: in the case of the chronomodulated delivery, beads retained their structure for the first 6 h, followed by swelling and erosion of CSSC, which occurred with the release of MP.

### 3.8. In Vivo Pharmacokinetic Studies

**Figure 10:**
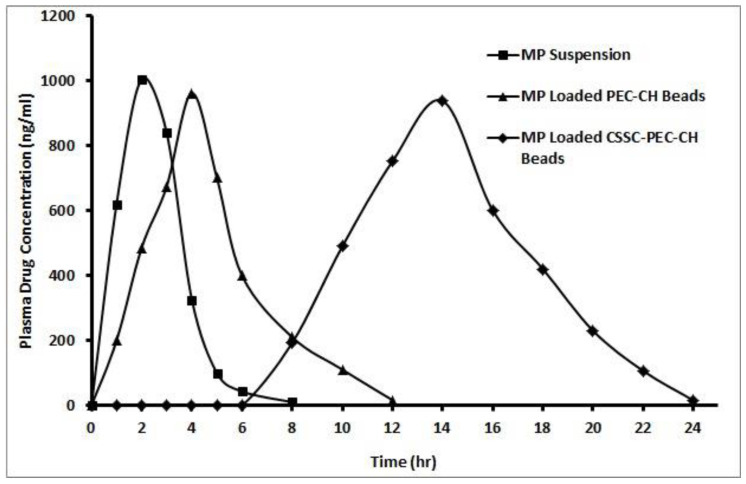
In vivo plasma concentration of MP from pure drug suspension, PEC-CH beads, and CSSC-coated PEC-CH beads.

The plasma drug release concentration versus time plot for the free drug and the drug released from its optimum chronomodulated formulation is illustrated in Figure 10. HPLC results of rat plasma samples of pure medication, PEC-CH beads, and optimized chronomodulated CSSC-PEC-CH beads were used to elicit several pharmacokinetic parameters, as shown in Table 4. There were no statistically significant changes seen in the AUC last and C_max_ values of the PEC-CH beads and CSSC-PEC-CH bead formulations (*p* < 0.001) (Table 3). There were no statistically significant changes seen in the AUC last and C_max_ values of the PEC-CH beads and CSSC-PEC-CH bead formulations (*p* < 0.001) (Table 4) [33]. From these, it was found that a similar quantity of drug was available in all formulations investigated and the application of CSSC coating did not considerably impact the bioavailability of MP. The amino group of chitosan (NH_3_^+^) and the carboxyl group of pectin (COO^−)^ come into electrostatic contact to form PEC-CHs. Pectin and chitosan may also interact through hydrogen bonding, providing enhanced hydrophilicity and causing swelling in PEC-CH-based hydrogels, resulting in an increase in porosity and, consequently, an increase in load diffusion. This explains why PEC-CH beads released the drug at a lower pH. CSSC-coated beads protect the medication release at a lower pH and release in a neutral pH environment after reaching colonic pH [34,35]. The positively charged main amino group contained in the chitosan backbone chain interacts with the negative location of the tight junction and the consequent enlargement of the paracellular transport route will facilitate the gastrointestinal absorption of MP. There is a substantial increase in T_max_, AUC, and MRT of the optimized chronomodulated system of CSSC-PEC-CH beads compared with that of pure medication found by pharmacokinetic analysis. The study established a time-delayed drug release with better MP pharmacokinetic properties from its optimized chronomodulated delivery systems. CSSC-PEC-CH beads were utilized to accomplish MP chronopharmacokinetics, which were found to be reasonably stable in the gastrointestinal system across time.

As a result, effective chronomodulated MP delivery was obtained. The lag phase generated by the chitosan succinate polymer’s pH-dependent solubility qualities in the stomach is what caused the T_max_ to be attained at 10 h in the in vivo research of the CSSC-coated PEC-CH beads.

### 3.9. Histopathological Studies

**Figure 11:**
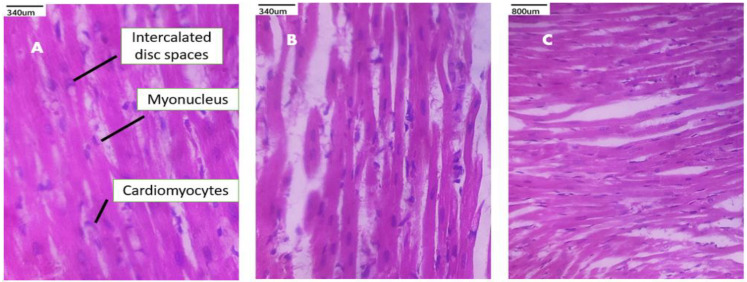
Histopathological images of heart sections (40×) show after treatment (**A**) MP suspension; (**B**) PEC-CH beads; (**C**) CSSC-coated PEC-CH beads.

**Figure 12:**
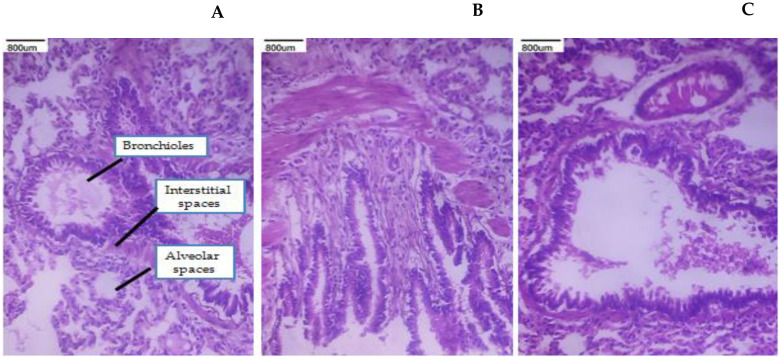
Histopathological images of lung sections (40×) show after treatment. (**A**) MP suspension; (**B**) PEC-CH beads; (**C**) CSSC-coated PEC-CH beads.

**Figure 13:**
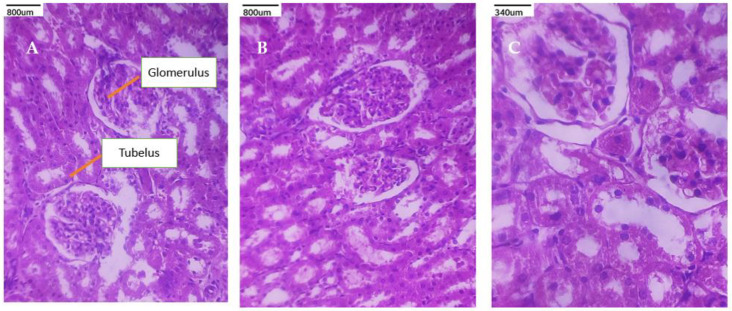
Histopathological images of kidney sections (40×) show after treatment. (**A**) MPsuspension; (**B**) PEC-CH beads; (**C**) CSSC-coated PEC-CH beads.

A toxicity assessment was conducted on the lung, kidney, and heart histopathology examinations in order to evaluate the MP suspension, PEC-CH beads, and PEC-CH beads coated with CSSC. The corresponding figures display the tissue toxicity images. It was seen from the figures that the histological scans of the lungs showed normal alveolar gaps and bronchioles. The kidney’s histological pictures show that the tubules and glomerular structures are normal. Cardiomyocytes with intact nuclei and a proper intercalated disc space were visible in heart slices. All of these results demonstrated that CSSC-coated PEC-CH beads of MP are safe and harmless for consumption by rats [21,36,37,38,39,40]. The histopathological studies are shown in Figure 11, Figure 12 and Figure 13.

### 3.10. Stability Studies of MP CSSC-PC-CH Nanoparticles

The assay of MP, lag time to release (T), and bead size did not significantly alter after six months of storage, suggesting that the formulation is both physically stable and resilient to handling and storage-related variations in ambient temperature and humidity. When stability data from the original samples and the stored samples were compared, this was verified (Table 5).

## 4. Conclusions

The present study successfully developed and characterized a chronomodulated nanoparticulate delivery system for MP, designed to target nocturnal asthma. Chitosan and pectin were selected through computational simulations as optimal polymers due to their biocompatibility, mucoadhesive properties, and ability to enable controlled release when coated with CSSC. The CSSC coating facilitated pH-sensitive release, protecting MP in the stomach and ensuring targeted delivery in the small intestine, synchronizing with early morning asthma exacerbations. Both in vitro and in vivo analyses demonstrated that CSSC-PEC-CH beads provided stable and sustained drug release over 24 h, significantly enhancing MP bioavailability in rat models. Histopathological evaluations confirmed the formulation’s nontoxicity, supporting its clinical safety. Stability testing showed that the CSSC-coated formulation maintained its efficacy under various storage conditions. The CSSC-PEC-CH nanoparticulate delivery system offers a promising platform for chronomodulated drug delivery aimed at addressing nocturnal asthma.

## Figures and Tables

**Table 1:** Composition of MP-loaded CSSC-PEC-CH beads for chronomodulated delivery.

Code	Formulation	MP (1%)	CH (%)	PEC (%)	CSSC (%)	CaCl_2_ (%)
F1	CH NP	1	1	2	-	-
F2	PEC-CH NP	1	1	2	-	10
F3	PEC-CH NP	1	1	2	1.0	10
F4	PEC-CH NP	1	1	2	1.5	10
F5	PEC-CH NP	1	1	2	2.0	10

**Table 2:** Energies of drug and polymer after the Forcite minimization.

Drug and Polymer Name	Initial Structure (Total Energy)	Final Structure (Total Energy)	Initial Structure (RMS Force)	Final Structure (RMS Force)
MP	80,572,880.000	85,895	6.650 × 10^17^	1.286 × 10^−2^
Starch	327,479,148.90	59,071	8.805 × 10^9^	1.877 × 10^−4^
Sodium Alginate	102,186.851	23,576	1.149 × 10^5^	1.756 × 10^−4^
Pectin	224,998.84	12,242	4.825 × 10^17^	8.946 × 10^−5^
Lactose	3,274,791.906	59,071	8.805 × 10^9^	1.877 × 10^−4^
HPMC	1,695,365.320	256,425	3.012 × 10^11^	1.457 × 10^0^
Ethylcellulose	6,710,585.977	81,746	1.047 × 10^8^	7.931 × 10^−3^
Chitosan	8272841.958	301,128	6.495 × 10^9^	6.084 × 10^−1^
Carboxymethyl Cellulose	1,553,603.007	13,431	1.036 × 10^12^	3.680 × 10^−1^

All energies are expressed in kcal/mol. All force is expressed in kcal/mol/A.

**Table 3:** Mixing energy values of the drug–polymer combination at a constant 298 k temperature.

Base	Screen	Avg Base-Screen Mix (−298 K)	E-Mix	Adsorption Energy
MP	Starch	−5.40755618	2.96487303	−32.79969
MP	Sodium Alginate	−3.37076904	7.84406254	−29.631640
MP	Pectin	−3.12717716	8.69480618	−29.736924
MP	Lactose	−5.33106282	3.40672579	−32.8891896
MP	HPMC	−9.44170033	17.81168398	−65.1071738
MP	Ethyl cellulose	−7.27462157	3.33341370	−39.903065
MP	Chitosan	−11.48541631	−8.99686240	−55.169463
MP	Carboxymethyl Cellulose	−3.92782055	6.66274535	−34.3474747

**Table 4:** Chronopharmacokinetic parameters of CSSC-coated PEC-CH beads.

Pharmacokinetic Parameters	MP	MP-Loaded PEC-CH Beads	MP-Loaded CSSC-Coated PEC-CH Beads
C_max_ (ng/mL)	1006.8 ± 32.68	963.11 ± 24.5	939.2 ± 20.11
T_max_ (h)	2 ± 1	4 ± 1	10 ± 1
AUC_0–t_ (ng/mL × h)	2980 ± 52.33	4032.71 ± 79.33	7298.48 ± 64.07
AUMC_0–t_ (ng/mL × h^2^)	3278.23 ± 22.59	16,853.76 ± 26.58	60,281.48 ± 0.52
MRT (h)	1.10 ± 0.59	4.18 ± 1.20	8.26 ± 2.34
Relative Bioavailability (%)	_	135.30 ± 7.6	244.89 ± 6.78 *

* p < 0.001.

**Table 5:** Stability study report of CSSC-coated PEC-CH beads at 25 °C/60% RH and 40 °C/75% RH.

Storage Temperature Condition	Beads Size (µm)		Drug Content (%)		Lag Time to Release (T_lag_) (h)	
	Initial	After Six Months	Initial	After Six Months	Initial	After Six Months
25 °C/60% RH	0.98 ± 0.21	0.96 ± 0.30	99.38 ± 1.72	98.65 ± 1.90	6.0 ± 0.15	6.0 ± 0.62
40 °C/75% RH		0.98 ± 0.43		95.87 ± 2.56		6.0 ± 0.97

## Data Availability

Data are contained within the article.
